# Melatonin Improves H_2_O_2_-Induced Oxidative Stress in Sertoli Cells Through Nrf2-Keap1 Signaling Pathway

**DOI:** 10.3390/genes15121544

**Published:** 2024-11-28

**Authors:** Ying Tang, Ziming Wang, Yanru Chen, Junying Wang, Hongzhan Wang, Bo Li, Bojing Liu, Peng Zheng

**Affiliations:** Department of Animal Genetics, Breeding and Reproduction, College of Animal Science and Technology, Northeast Agricultural University, Harbin 150030, China; 15905233543@163.com (Y.T.); zh-96128@163.com (Z.W.); yanruchen1012@gmail.com (Y.C.); 13325157620@163.com (J.W.); 18937298367@163.com (H.W.); 15161330947@163.com (B.L.); 18632847220@163.com (B.L.)

**Keywords:** Sertoli cells, melatonin, hydrogen peroxide, oxidative stress, Nrf2-Keap1

## Abstract

**Background**: Oxidative stress in the testicles of male livestock can cause reduced fertility. Melatonin is a natural product with antioxidant effects, but its specific antioxidant mechanism is still unclear. This study used calf testicular Sertoli cells as materials to explore the mechanism by which melatonin alleviates the oxidative stress of Sertoli cells, laying a foundation for improving the fertility of bulls. **Methods**: The optimal treatment concentrations of H_2_O_2_ and melatonin (MLT) were screened out using a CCK8 kit and MDA kit. Then, the cells were divided into four groups for treatment: control group, H_2_O_2_ treatment group, MLT treatment group, and H_2_O_2_ and MLT co-treatment group, then the MDA, ROS, GSH, and SOD contents were detected. Real-time quantitative PCR analysis and Western blot analysis were used to detect genes and proteins related to the Nrf2-Keap1 pathway. Immunofluorescence staining was used to analyze changes in Nrf2. **Results**: Research results show that the MDA content of cells in the group treated with H_2_O_2_ and MLT combined was significantly lower than that in the group treated with H_2_O_2_ alone, but there was no difference from the control group. Compared with the control group, the ROS level of cells in the H_2_O_2_-treated group significantly increased, and the content of GSH and SOD significantly decreased. Compared with the H_2_O_2_-treated group, the ROS level of cells in the H_2_O_2_ and MLT co-treated group significantly decreased, and the content of GSH and SOD increased significantly, but no difference from the control group. Similarly, MTL can alleviate the changes in cellular Nrf2, Keap1, HO-1, and NQO1 expression caused by H_2_O_2_. **Conclusions**: Melatonin activates the Nrf2-Keap1 signaling pathway in Sertoli cells, elevating the expression of HO-1 and NQO1, and thereby exerting its antioxidant capabilities.

## 1. Introduction

In livestock production, oxidative stress (OS) is an important factor leading to male livestock infertility [1]. In vivo experiments in sheep found that OS can significantly inhibit the synthesis of testosterone, leading to loss of fertility [2]. In pigs, OS reduces the mitochondrial membrane potential in pig testes and significantly promotes apoptosis and autophagy of testicular cells [3]. In chickens, OS will cause the semen quality of breeding males to decrease, directly affecting the fertilization rate of hatching eggs and the quality of chicks [4].

The generation of OS is mainly due to the increase of ROS in the body. As a highly reactive small molecule, reactive oxygen species (ROS) are usually short-lived. Trace amounts of ROS play a key role in regulating normal physiological functions such as cell cycle progression, proliferation, migration, cell death, and maintaining redox homeostasis [5]. On the contrary, high concentrations of ROS are harmful and can induce OS. At the cellular level, oxidative stress caused by physical or chemical factors activates a series of reactions in the body, thereby inducing the expression of a series of cell protection products, such as superoxide dismutase (SOD), glutathione peroxidase (GSH-Px), and catalase (CAT). Li showed that SOD is the first line of defense against oxidative stress [6]. Studies on mouse Leydig cells have found that increased SOD gene expression levels can significantly reduce the number of late-stage apoptotic TM3 cells; interfering with the activity of the antioxidant enzyme SOD can cause the accumulation of lipid peroxide malondialdehyde (MDA) in the body, increase the content of oxygen-free radicals, induce OS in TM3 cells, and aggravate cell damage [7]. GSH-Px is a selenoprotein that can specifically and effectively remove phospholipid hydroperoxides and can also eliminate excess ROS by consuming glutathione (GSH) [6]. Studies have shown that these antioxidant enzymes can be expressed in the testis [8]. Hydrogen peroxide (H_2_O_2_), as a strong oxidant, can freely pass through the cell membrane and react with intracellular Fe^3+^ to produce hydroxyl free radicals, thereby causing OS in the body [9]. In addition, H_2_O_2_ is stable in nature, not easily decomposed in the body, and widely available. It has become a commonly used reagent in inducing animal tissue or cell oxidative damage models [10].

Sertoli cells (SCs) are important somatic cells in the testis. They can form seminiferous tubules with germ cells and myoid cells. They are closely related to spermatogenesis and are also crucial to the maturation of germ cells [11]. Experimental studies have shown that androgen synthesis in SCs of mice with knockout androgen receptor genes is disordered, resulting in the inability of germ cells to complete meiosis and ultimately disrupted spermatogenesis [12]. SCs also synthesize and secrete many substances required for the spermatogenesis process, such as androgen binding protein (ABP). ABP locally controls the proliferation, maturation, and participation of spermatogenic cells in spermatogenesis [13].

Melatonin (MLT) is closely related to rhythm regulation, anti-inflammation, sexual maturation, and other effects [14]. In mouse testicular Leydig cells, melatonin can protect mouse cells from oxidative stress by activating the Sirt1/Nrf2 signaling pathway [15]. In porcine SCs, MLT can alleviate CYP-induced oxidative stress, DNA damage, and apoptosis. In porcine Sertoli cells, melatonin can protect them from oxidative damage by regulating Nrf2 activity [16]. In addition, melatonin is also a powerful antioxidant. Nowadays, there are many studies on the antioxidant aspect of MLT, and it has been used in different species. In mouse testicular Leydig cells, MLT pretreatment can alleviate heat-induced oxidative stress and apoptosis in the testis, thereby allowing the testis to recover from heat injury [17]. In porcine SCs, MLT can alleviate CYP-induced oxidative stress, DNA damage, and apoptosis in porcine SCs [18]. Zhu et al. have shown that adding MLT to freezing additives can significantly increase the activity of GSH and SOD and reduce the content of ROS, thereby protecting rabbit sperm from OS. Dong et al. studied that MLT alleviated oxidative stress in rooster Leydig cells by reducing ROS, MDA levels and increasing SOD and GSH-Px activities [19]. However, there are currently few reports on the antioxidant mechanism of melatonin in bovine testicular Sertoli cells.

Therefore, this study first used H_2_O_2_ to induce oxidative stress in calf Sertoli cells, and then injected melatonin into the Sertoli cells under oxidative stress to explore the antioxidant properties of melatonin in calf Sertoli cells and its regulatory mechanism.

## 2. Materials and Methods

### 2.1. Reagents and Chemicals

Bovine Sertoli cells were previously isolated, purified, and stored in our laboratory [20]. We utilized the following specific reagents and kits for our research, including Trizol^®^ (Cat. no.: 15596026, Invitrogen, Shanghai, China), CCK-8 kit (Cat. no.: B34304, Bimake Company, Beijing, China), fetal bovine serum (Cat. no.: P30-3302, PAN-Biotech, Adenbach, Germany), a reverse transcription kit (Cat. no.: RR047A, Takara, Beijing, China), ROX (Cat. no.: 72986700, Roche, Shanghai, China), and BCA Protein Assay kit (Cat. no.: P0011), MDA kit (Cat. no.: E003-1-1), GSH kit (Cat. no.: E006-1-1), SOD kit (Cat. no.: A001-1-1), and ROS kit (Cat. no.: E004-1-1) were purchased from China Nanjing Jiancheng Bioengineering (Nanjing, China). Additionally, we used DMEM/F12 (Cat. no.: D8437, Sigma, Shanghai, China) and 3% H_2_O_2_.

### 2.2. Cell Culture and Treatment

The Sertoli cell culture and treatment procedures followed our previously established protocols. In brief, cells were thawed and passaged into a 60 mm culture dish. When cell growth reached over 80%, they were subjected to treatments with H_2_O_2_ and melatonin. The concentrations of H_2_O_2_ used were 0, 50, 100, and 300 µmol/L, while the melatonin concentrations were 0, 0.1, 1, and 10 µmol/L, respectively. Through CCK8 and MDA content determination, the lowest concentration that affects Sertoli cell viability and MDA content was screened out, thus serving as the optimal concentration of H_2_O_2_ and MLT. Based on the screening results, the cells were categorized into four treatment groups: control group, H_2_O_2_ treatment group (300 µmol/L H_2_O_2_), melatonin treatment group (1 µmol/L melatonin), H_2_O_2_ and melatonin co-treatment group (300 µmol/L H_2_O_2_ + 1 µmol/L melatonin). Subsequently, the treated cells underwent further analysis and evaluation.

### 2.3. Cell Viability Assay and MDA Determination

The cell viability of calf testicular support cells was measured using the CCK8 kit. The cells were inoculated in a 96-well plate (10^4^ cells per well) and cultured for 12 h, then treated with different concentrations of H_2_O_2_ and MLT, and blank wells (containing only culture medium) were set up. After 24 h, 10 µL CCK8 reagent was added, incubated at 37 °C for 2 h, and the *D*_450nm_ value was measured. Repeat 3 times for each treatment group.

Use the MDA kit to measure the MDA content and prepare the working solution according to the instructions. After treating the cells with different concentrations of H_2_O_2_ and MLT, wash the cells 3 times with PBS, then digest them with trypsin, collect the obtained cell suspension into a 1.5 mL EP tube, and use a centrifuge for centrifugation, remove the supernatant, and obtain the precipitate. Then, use an ultrasonic crusher to crush, add MDA working solution, mix well, incubate at 37 °C for 20 min, and use an enzyme marker to measure the *D*_450nm_ value. Finally, calculate according to the formula in the instructions. Repeat 3 times for each treatment group.

### 2.4. The Detection of ROS Production, GSH Content, and SOD Activity

The ROS content was detected by DCFH-DA staining. The cells were inoculated into a 60 mm culture dish and cultured for 12 h. Then, the cells were divided into a control group, an H_2_O_2_ treatment group (300 µmol/L H_2_O_2_), a melatonin treatment group (1 µmol/L melatonin), and an H_2_O_2_ and melatonin combined treatment group (300 µmol/L H_2_O_2_ + 1 µmol/L melatonin). The treatment continued for 24 h. Then, 1–2 mL of 10 µmol/L DCFH-DA was added, and incubated at 37 °C in the dark for 30 min. Pre-cooled PBS was rinsed repeatedly 3 times, and then fluorescence detection was performed.

GSH and SOD kits were used to detect the content of GSH and SOD, and then the cells were divided into a control group, an H_2_O_2_ treatment group (300 µmol/L H_2_O_2_), a melatonin treatment group (1 µmol/L melatonin), and combined H_2_O_2_ and melatonin treatment group (300 µmol/L H_2_O_2_ + 1 µmol/L melatonin), and the treatment was continued for 24 h. The cells were repeatedly rinsed with PBS 3 times and then digested with trypsin. The obtained cell suspension was collected in a 1.5 mL EP tube. Using a centrifuge to obtain the precipitation. Subsequently, an ultrasonic disruptor was used for disruption, and the GSH working solution was added, mixed, and incubated at 37 °C for 20 min. The *D*_405nm_ value was measured; the SOD working solution was added, mixed, and incubated at 37 °C for 20 min, and the *D*_450nm_ value was measured. Finally, the calculation was performed according to the formula in the instructions. Each treatment group was repeated 3 times.

### 2.5. Real-Time Quantitative PCR Analysis

Total RNA was extracted using Trizol^®^ reagent. The reverse transcription process was carried out according to the instructions. Subsequently, the diluted cDNA served as a template. The PCR reaction system was 10 μL: FastStart Universal SYBR Master (ROX) 5 μL, forward primers and reverse primers (10 μmol/L) 0.3 μL each, cDNA 1 μL, RNase-free ddH_2_O 3.4 μL. PCR reaction program: 95 °C pre-denaturation for 30 s; 95 °C denaturation for 15 s, 60 °C annealing for 60 s, for a total of 40 cycles. The β-actin gene was used as an endogenous control and the target gene expression level was calculated using the 2^−ΔΔCT^ method. The BGI gene company in China synthesized all primers, and you can find the details of primer sequences in Table 1.

### 2.6. Western-Blot Analysis

To prepare the samples, we started by adding 100 µL of RIPA lysis buffer and 4 µL of 25× protease inhibitor to the collected cells. This allowed us to extract intracellular proteins, which were subsequently quantified using the BCA protein assay kit. The protein samples were then uniformly diluted with PBS. Next, we subjected the proteins to denaturation and electrophoresis, followed by their transfer onto a PVDF membrane. The membrane was sealed with 5% skim milk powder at room temperature for 2 h. After sealing, we followed the antibody manufacturer’s instructions (rabbit anti-Nrf2, rabbit anti-Keap1, rabbit anti-HO-1, rabbit anti-NQO1, rabbit anti-Lamini B1 and β-actin, 1:1000, Bioss, Shanghai, China) and incubated the membrane overnight at 4 °C. Washing 3 times with PBS for 5 min each time. Subsequently, we applied the secondary antibody (HRP Goat Anti-Rabbit IgG H&L, 1:10,000, Bioss, Shanghai, China) and incubated it at room temperature for 2 h. The membrane was washed three more times with TBST for 5 min each. The ECL Western blotting system was used for exposure and photography. Image J 1.8.0 software was used for band densitometry analysis. For reliability, this experiment was repeated three times for each treatment group. To assess the nuclear translocation of the nuclear factor erythroid 2-related factor 2 (Nrf2) protein, we employed nuclear and cytoplasmic protein extraction kits to isolate the proteins, followed by adherence to the Western blot experimental protocol.

### 2.7. Immunofluorescence

First, cells were fixed for 10 min using 4% paraformaldehyde and 0.2% Triton X-100. After fixation, 3 rinses were performed using PBS, followed by blocking in 1% BSA for 30 min. Subsequently, the rabbit anti-Nrf2 antibody (1:100, Bioss, Shanghai, China) was added, and the cells were incubated with this antibody overnight at 4 °C. The following day, the secondary antibody (Goat Anti-rabbit IgG H&L/FITC, 1:500, Bioss, Shanghai, China) was added for 2 h at room temperature. The nuclei were stained using DAPI. Fluorescent images were captured using a confocal microscope (Nikon, Tokyo, Japan). All morphological measurements were conducted in triplicate.

### 2.8. Statistical Analysis

Experimental data were analyzed using SPSS 21.0 software and Excel 2020 worksheet, and GraphPad Prism 8.3 was used for drawing. Data analysis adopts a one-way analysis of variance, and test evaluation adopts the Tukey method. All data are expressed as mean ± SD. *p* < 0.05 was considered significant.

## 3. Results

### 3.1. Effects of H_2_O_2_ and Melatonin on Cell Viability and MDA Content

**Figure 1:**
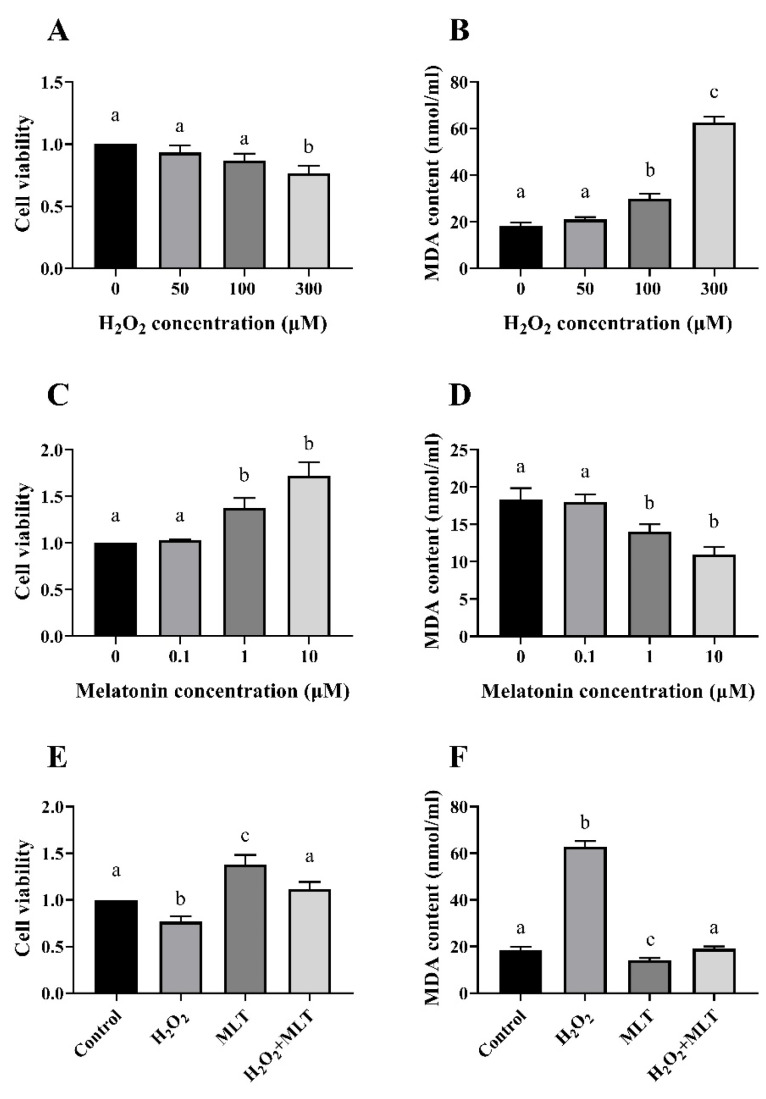
Effects of different concentrations of H_2_O_2_ and MLT on cell viability and MDA. (**A**) Effect of H_2_O_2_ on cell viability; (**B**) Effect of H_2_O_2_ on the content of MDA in cells; (**C**) Effects of MLT on cell viability; (**D**) Effect of MLT on the content of MDA in cells; (**E**) Changes in cell viability; (**F**) Changes in MDA content. Control group, H_2_O_2_ group (300 µmol/L H_2_O_2_), MLT group (1 µmol/L MLT), H_2_O_2_ + MLT group (300 µmol/L H_2_O_2_ + 1 µmol/L MLT). Different letters on the shoulder mark indicate significant differences (*p* < 0.05); Markings with the same letter on the shoulder mark indicate that the difference is not significant (*p* > 0.05).

Compared with the control group, the viability of cells treated with 300 µmol/L H_2_O_2_ decreased significantly (Figure 1A). The MDA content of cells treated with 100 µmol/L and 300 µmol/L H_2_O_2_ was significantly increased (Figure 1B). The viability of cells treated with 1 µmol/L and 10 µmol/L MLT was significantly increased (Figure 1C). The MDA content of cells treated with 1 µmol/L and 10 µmol/L MLT decreased significantly (Figure 1D). Therefore, 300 µmol/L H_2_O_2_ and 1 µmol/L MLT were selected for further experiments. After co-treatment with H_2_O_2_ and MLT, the cell viability was significantly higher than that of H_2_O_2_ treatment, substantially lower than that of MLT treatment, and not considerably different from the control group (Figure 1E); the MDA content was significantly lower than that of H_2_O_2_ treatment, and fundamentally higher than that of MLT treatment, there was no substantial difference from the control group (Figure 1F).

### 3.2. The Effects of H_2_O_2_ or/and MLT on ROS, GSH, and SOD in SCs

**Figure 2:**
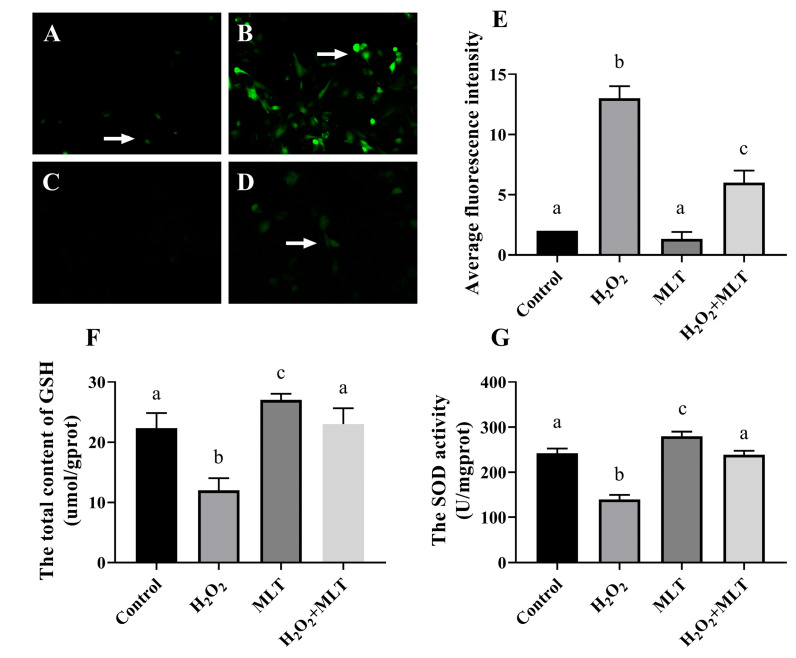
Effects of different concentrations of H_2_O_2_ and MLT on ROS, GSH, and SOD. (**A**–**D**) ROS staining results, the arrow indicates positive cells; (**A**) Control group; (**B**) H_2_O_2_ group; (**C**) MLT group; (**D**) H_2_O_2_ + MLT group. (**E**) ROS results analysis; (**F**) Changes in GSH activity; (**G**) Changes in SOD activity. Different letters on the shoulder mark indicate significant differences (*p* < 0.05); Markings with the same letter on the shoulder mark indicate that the difference is not significant (*p* > 0.05).

The results of ROS staining are shown in Figure 2A–D. Observation under a fluorescence microscope revealed that the H_2_O_2_ group showed a large amount of green fluorescence (Figure 2B). This means that a large amount of reactive oxygen species (ROS) is produced in the cells. In contrast, in the H_2_O_2_ and MLT co-treatment group (Figure 2D), green fluorescence disappeared and the level of ROS was reduced when compared with the H_2_O_2_ group (Figure 2B). Additionally, the histogram representing the average fluorescence intensity values (Figure 2E) revealed that the average fluorescence intensity in the H_2_O_2_ group was notably higher than that of the other three groups (*p* < 0.05). However, the average fluorescence intensity in the H_2_O_2_ and MLT co-treatment group was lower compared to the H_2_O_2_ group (*p* < 0.05). Similarly, the cellular content of GSH and SOD activity in the H_2_O_2_ group was significantly lower than those in the control group. In contrast, the cellular GSH content and SOD activity in the H_2_O_2_ and MLT co-treatment group were significantly higher than those in the H_2_O_2_ group (Figure 2F,G), there was no significant difference from the control group.

### 3.3. The Effect of H_2_O_2_ or/and MLT on the Keap1, HO-1, and NQO1

**Figure 3:**
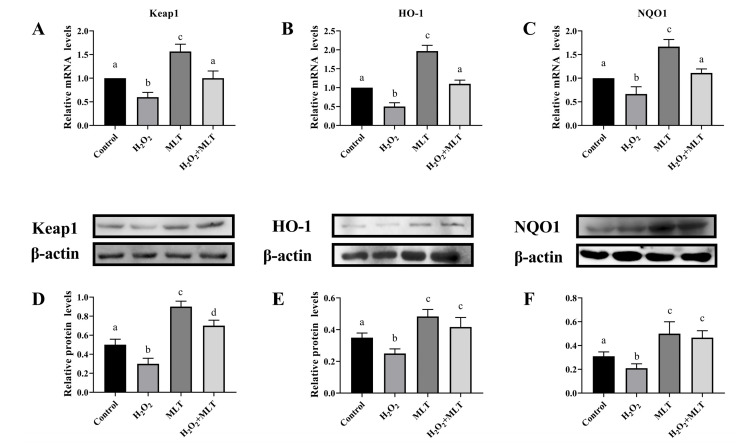
The level of expression of Keap1, HO-1, and NQO1 gene mRNA. (**A**–**C**) effects on Keap1, HO-1 and NQO1 mRNA expression in SCs. (**D**–**F**) effects on Keap1, HO-1 and NQO1 protein expression in SCs. Different letters on the shoulder mark indicate significant differences (*p* < 0.05); Markings with the same letter on the shoulder mark indicate that the difference is not significant (*p* > 0.05).

As shown in Figure 3, when compared to the control group, the mRNA and protein expression of Kelch-like ECH-associated protein 1 (Keap1), hemeoxygenase-1 (HO-1), and recombinant NADH Dehydrogenase Quinone 1 (NQO1) exhibited a significant decrease following H_2_O_2_ treatment (*p* < 0.05), the mRNA and protein expression of Keap1, HO-1, and NQO1 exhibited a significant increase following MLT treatment (*p* < 0.05). On the other hand, after H_2_O_2_ and MLT treatment, the mRNA and protein expression of Keap1, HO-1, and NQO1 showed a significant increase when compared to the H_2_O_2_ group (*p* < 0.05), and there was no significant difference from the control group.

### 3.4. The Effect of H_2_O_2_ or/and MLT on Nrf2

**Figure 4:**
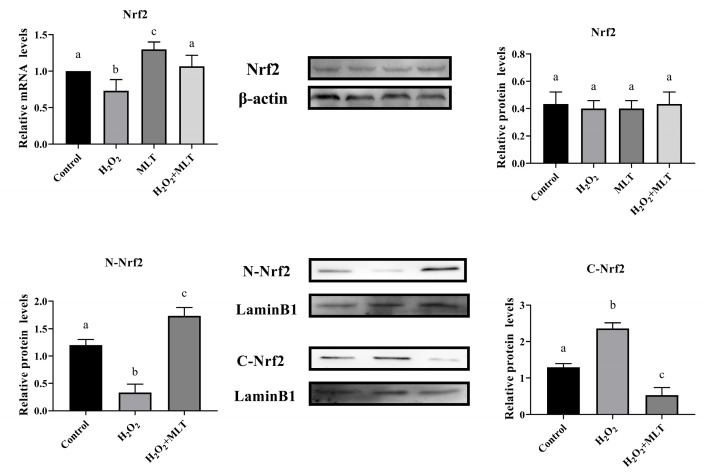
Effect of melatonin on nuclear translocation of Nrf2 protein in calf Sertoli cells under oxidative stress. Different letters on the shoulder mark indicate significant differences (*p* < 0.05); Markings with the same letter on the shoulder mark indicate that the difference is not significant (*p* >0.05).

The results of qPCR and Western blot analysis are shown in the figure. When compared to the control group, the mRNA expression of Nrf2 exhibited a significant decrease following H_2_O_2_ treatment (*p* < 0.05), and Nrf2 mRNA expression was significantly increased following MLT treatment (*p* < 0.05). After H_2_O_2_ and MLT treatment, the mRNA expression of Nrf2 showed a significant increase when compared to the H_2_O_2_ group (*p* < 0.05), there was no significant difference from the control group. However, across all experimental groups, there were no significant changes observed in the protein levels of Nrf2. Therefore, in the following experiments, we detected the expression of nuclear Nrf2 protein and cytoplasmic Nrf2 protein (Figure 4).

**Figure 5:**
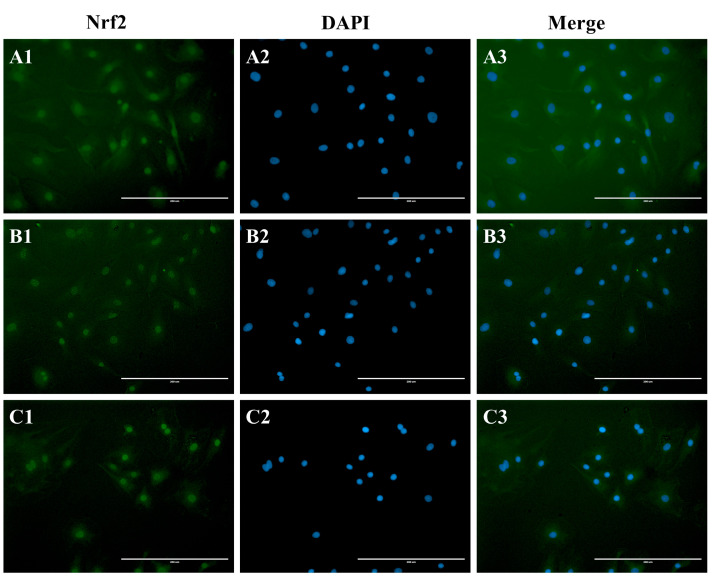
Effects of melatonin on Nrf2 protein expression. (**A**) Control group, (**B**) H_2_O_2_ group, (**C**) MLT + H_2_O_2_ group.

Immunofluorescence test results are shown in the figure (Figure 5). Across all experimental groups, the Nrf2 protein was found in both the cytoplasm and the nucleus. However, in comparison to the control (Figure 5A) and the H_2_O_2_ groups (Figure 5B), the co-treatment with melatonin and H_2_O_2_ (Figure 5C) led to a significant enhancement in the intensity of green fluorescence within the nucleus.

## 4. Discussion

In recent years, SCs have also become model cells for studying male reproductive toxicology in vitro [21]. Active oxygen accumulated in the reproductive system can cause abnormal biochemical reactions in germ cells, destruction of organelles, breakage of DNA monotonic structure, abnormal repair, and other reactions, leading to apoptosis and even necrosis [22]. Therefore, this experiment used H_2_O_2_ and melatonin to treat calf testicular Sertoli cells to study the protective effect of melatonin on H_2_O_2_-induced oxidative stress in calf testicular SCs. The specific conditions for inducing cellular oxidative stress may vary slightly for different cell types, but in general, they typically involve reducing cellular activity to a range of 70%. If the activity level is higher, it may not effectively cause oxidative stress, while excessively low activity can lead to significant cellular damage and result in mass cell death [23]. MDA is the main product of membrane lipid peroxidation, which is harmful to the body and is usually the most important indicator for measuring lipid peroxidation [24]. MDA, in cells, can cause deformations and cross-linking of proteins and phospholipids, leading to the contraction or shrinking of the cell membrane. The MDA content can indirectly reflect the degree of cell invasion and the degree of fat loss [22]. Hence, this study opted to assess the concentration and exposure duration of H_2_O_2_ and melatonin by examining MDA levels and cell viability within the cells. The results revealed that the MDA content in the H_2_O_2_ group was notably higher than that in the control group, signifying that the cells were indeed under oxidative stress at this point. Consequently, this experiment successfully established and validated the oxidative stress model. We used different concentrations of melatonin for treatment, and the results showed that 1 µmol/L of melatonin significantly increased cell viability and reduced MDA content, which is consistent with the results of Li et al. [25]. Melatonin can improve cell vitality. In summary, the preliminary research results found that melatonin can promote the growth of cells under oxidative stress. Furthermore, in comparison to the group treated solely with H_2_O_2_, the count of ROS-positive cells noticeably decreased, and intracellular GSH and SOD levels significantly increased following the combined treatment of melatonin and H_2_O_2_. This suggests that melatonin effectively hinders the accumulation of ROS within cells, improving their capacity to withstand oxidative stress.

The Nrf2-Keap1 signaling pathway is the main antioxidant response regulatory pathway in the body [26]. It has the ability to resist free radical damage, promote the expression of multiple antioxidant enzyme genes, and maintain the body’s redox balance [27]. Nrf2 is a key transcription factor. Under normal physiological conditions, Nrf2 is mainly distributed in the cytoplasm and is coupled to its inhibitory protein Keap1. When the body is stimulated by reactive oxygen species (ROS), electrophilic reagents, etc., Nrf2 is degraded by the ubiquitination proteasome, and only a small part of stable Nrf2 dissociates from Keap1 and is transferred to the nucleus to play its role [28]. In the presence of antioxidant substances, Nrf2 dissociates from Keap1, promotes Nrf2 transport to the nucleus, and binds to the regulatory protein (Maf) in the nucleus to promote the transcription of downstream target genes [29]. This is consistent with our results. The mRNA expression of Nrf2 was significantly reduced after H_2_O_2_ treatment (*p* < 0.05), and the mRNA expression of Nrf2 was significantly increased after MLT treatment (*p* < 0.05). In this experiment, there was no significant difference in the relative expression level of Nrf2 protein in each treatment group. This phenomenon may be due to the fact that the Nrf2-Keap1 signaling pathway needs to be carried out under the condition that Nrf2 is separated from Keap1 so that Nrf2 can enter the nucleus and activate the downstream target gene sequence. Therefore, in follow-up studies, Western blot technology was used to monitor changes in the content of Nrf2 (N-Nrf2) in the nucleus and cytoplasm (C-Nrf2), and immunofluorescence technology was used to locate Nrf2. The results showed that in Western blot detection, the expression level of Nrf2 protein in the nucleus of the combination group was higher than that in the H_2_O_2_ group after H_2_O_2_ treatment, while the expression level in the cytoplasm was lower than that in the H_2_O_2_ group. In immunofluorescence detection, the localization of Nrf2 protein in the nucleus of the combined drug group was higher than that of the H_2_O_2_ group. Therefore, based on the results of this experiment, we believe that the pre-protective effect of melatonin may promote the nuclear translocation of Nrf2 protein to exert its antioxidant function, ultimately leading to changes in the expression level of Nrf2 protein between the nucleus and cytoplasm.

Among the many downstream proteins regulated by Nrf2, NQO-1, and HO-1 are the two most prominent and widely studied antioxidant proteins [30]. NQO-1 is a flavoprotein protease that can exert antioxidant effects. NADH or NADPH participates in the reaction as its electron donor and participates in the catalytic reaction with quinone and its derivatives, reducing its damage to cells, thereby preventing it from producing ROS and ensuring that cells are protected from oxidative stress caused by various metabolisms [31]. Studies by Estaras et al. have shown that melatonin can alleviate oxidative stress caused by hypoxia by regulating the level of NQO-1 in pancreatic stellate cells [32]. HO-1 is widely distributed in various tissues and organs throughout the body and has an antioxidant effect [33]. HO-1 protein is mainly located in the smooth endoplasmic reticulum and catalyzes the conversion of heme into bilirubin, carbon monoxide, and iron. Studies have also shown that HO-1 protein can increase the activity of multiple enzymes in nervous system cells, thereby protecting neural tissue from secondary damage [34]. Qin et al. found that melatonin can inhibit LPS-induced dendritic cell oxidative stress through the Nrf2-HO-1 axis [35]. This is consistent with our experimental results. The relative mRNA expression and protein expression of Keap-1, HO-1, and NQO1 in the H_2_O_2_ group showed the same trend, which was significantly different from that in the control group, indicating that the cells in the H_2_O_2_ group were affected by oxidative stress. The relative mRNA expression and protein expression of Keap1, NQO1, and HO-1 in the combined treatment group also showed a similar trend, which was significantly different from that in the H_2_O_2_ group. The combined treatment group enhanced the ability of cells to resist oxidative stress, indicating that melatonin (MLT) has a certain alleviating effect on H_2_O_2_-induced oxidative stress.

## 5. Conclusions

Melatonin has a protective effect on H_2_O_2_-induced oxidative stress in calf Sertoli cells. This protective effect is achieved by promoting the activation of the Nrf2-Keap1 signaling pathway, promoting the nuclear translocation of Nrf2, and increasing the activity and content of antioxidant enzymes in cells, thereby enhancing the antioxidant capacity of calf Sertoli cells.

## Figures and Tables

**Table 1:** Primer information for real-time quantitative PCR.

Genes	GenBank Accession No.	Primer Sequences (5′→3′)	Product Length/bp
NQO1	NM_001098104.1	F:CACTCTGCACTTCTGTGGCTTCC R:CAGGCGTTTCTTCCATCCTTCCAG	278
HO-1	NM_001046332.1	F:CCGCTACCTGGGAGACCTGTC R:ACTTGGTGGCACTGGCGATATTG	202
Nrf2	NM_001205732.1	F:TCAGCCAGCACAACACATACCATC R:ACGGGAATGTCTCTGCCAAAAGC	112
Keap1	NM_001002763.1	F:CGCCCTGGGAATTACCGTTCAC R:AACACTCCACGCTGTCCAAGAATG	387
β-actin	NM_173979.3	F:GCGGCATTCACGAAACTACCTT R:TCCTGCTTGCTGATCCACATCT	268

## Data Availability

None were deposited in an official repository. The data that support the study findings are available upon request.

## References

[1] Orrenius S., Nicotera P., Zhivotovsky B. (2011). Cell death mechanisms and their implications in toxicology. Toxicol. Sci..

[2] Lin L.-X., Cao Q.-Q., Zhang C.-D., Xu T.-T., Yue K., Li Q., Liu F., Wang X., Dong H.-J., Huang S.-C. (2022). Aflatoxin B1 causes oxidative stress and apoptosis in sheep testes associated with disrupting rumen microbiota. Ecotoxicol. Environ. Saf..

[3] Li Y., Chen H., Liao J., Chen K., Javed M.T., Qiao N., Zeng Q., Liu B., Yi J., Tang Z. (2021). Long-term copper exposure promotes apoptosis and autophagy by inducing oxidative stress in pig testis. Environ. Sci. Pollut. Res. Int..

[4] Escorcia M., Sánchez-Godoy F., Ramos-Vidales D., Medina-Campos O.N., Pedraza-Chaverri J. (2020). Effect of the Age and Body Weight of the Broiler Breeders Male on the Presentation of Oxidative Stress and Its Correlation with the Quality of Testicular Parenchyma and Physiological Antioxidant Levels. Vet. Sci..

[5] Zhang J., Wang X., Vikash V., Ye Q., Wu D., Liu Y., Dong W. (2016). ROS and ROS-Mediated Cellular Signaling. Oxid. Med. Cell Longev..

[6] Li J., Wang T., Liu P., Yang F., Wang X., Zheng W., Sun W. (2021). Hesperetin ameliorates hepatic oxidative stress and inflammation via the PI3K/AKT-Nrf2-ARE pathway in oleic acid-induced HepG2 cells and a rat model of high-fat diet-induced NAFLD. Food Funct..

[7] Lin R., Pian Y., Zhang C., Zhou L., Ren X. (2022). N-acetylcysteine alleviates cadmium-induced testicular interstitial cell apoptosis by activating protein kinase B pathway. Wei Sheng Yan Jiu.

[8] Turner T.T., Lysiak J.J. (2008). Oxidative stress: A common factor in testicular dysfunction. J. Androl..

[9] Warinhomhoun S., Muangnoi C., Buranasudja V., Mekboonsonglarp W., Rojsitthisak P., Likhitwitayawuid K., Sritularak B. (2021). Antioxidant Activities and Protective Effects of Dendropachol, a New Bisbibenzyl Compound from Dendrobium pachyglossum, on Hydrogen Peroxide-Induced Oxidative Stress in HaCaT Keratinocytes. Antioxidants.

[10] Oroojan A.A., Chenani N., An’aam M. (2020). Antioxidant Effects of Eugenol on Oxidative Stress Induced by Hydrogen Peroxide in Islets of Langerhans Isolated from Male Mouse. Int. J. Hepatol..

[11] Wang C., Zheng P., Adeniran S., Ma M., Huang F., Adegoke E., Zhang G. (2019). Thyroid hormone (T3) is involved in inhibiting the proliferation of newborn calf Sertoli cells via the PI3K/Akt signaling pathway in vitro. Theriogenology.

[12] Verhoeven G. (2005). A Sertoli cell-specific knock-out of the androgen receptor. Andrologia.

[13] Gao L., Gao D., Zhang J., Li C., Wu M., Xiao Y., Yang L., Ma T., Wang X., Zhang M. (2022). Age-related endoplasmic reticulum stress represses testosterone synthesis via attenuation of the circadian clock in Leydig cells. Theriogenology.

[14] Shi J.-F., Li Y.-K., Ren K., Xie Y.-J., Yin W.-D., Mo Z.-C. (2018). Characterization of cholesterol metabolism in Sertoli cells and spermatogenesis (Review). Mol. Med. Rep..

[15] Zhang J., Fang Y., Tang D., Xu X., Zhu X., Wu S., Yu H., Cheng H., Luo T., Shen Q. (2022). Activation of MT1/MT2 to Protect Testes and Leydig Cells against Cisplatin-Induced Oxidative Stress through the SIRT1/Nrf2 Signaling Pathway. Cells.

[16] BBartolini D., Arato I., Mancuso F., Giustarini D., Bellucci C., Vacca C., Aglietti M.C., Stabile A.M., Rossi R., Cruciani G. (2022). Melatonin modulates Nrf2 activity to protect porcine pre-pubertal Sertoli cells from the abnormal H_2_O_2_ generation and reductive stress effects of cadmium. J. Pineal Res..

[17] Zhu Z., Li R., Lv Y., Zeng W. (2019). Melatonin protects rabbit spermatozoa from cryo-damage via decreasing oxidative stress. Cryobiology.

[18] Li J., Sun B.-X., Wang D.-L., Liu Y., Qi J.-J., Nie X.-W., Bai C.-Y., Zhang J.-B., Liang S. (2021). Melatonin ameliorates cypermethrin-induced impairments by regulating oxidative stress, DNA damage and apoptosis in porcine Sertoli cells. Theriogenology.

[19] Dong Y., Zhao J., Zhu Q., Liu H., Wang J., Lu W. (2020). Melatonin inhibits the apoptosis of rooster Leydig cells by suppressing oxidative stress via AKT-Nrf2 pathway activation. Free Radic. Biol. Med..

[20] Wang X., Wang Z., Adeniran S.O., Huang F., Ma M., Zhang H., Li X., Zheng P., Zhang G. (2020). Wilms’ tumour 1 (WT1) negatively regulates the expression of connexin 43 via a non-canonical Wnt signalling pathway in cultured bovine Sertoli cells. Reprod. Fertil. Dev..

[21] Sakib S., Lara N.d.L.e.M., Huynh B.C., Dobrinski I. (2022). Organotypic Rat Testicular Organoids for the Study of Testicular Maturation and Toxicology. Front. Endocrinol..

[22] Zhang F., You X., Zhu T., Gao S., Wang Y., Wang R., Yu H., Qian B. (2020). Silica nanoparticles enhance germ cell apoptosis by inducing reactive oxygen species (ROS) formation in Caenorhabditis elegans. J. Toxicol. Sci..

[23] Chong C.-M., Zheng W. (2016). Artemisinin protects human retinal pigment epithelial cells from hydrogen peroxide-induced oxidative damage through activation of ERK/CREB signaling. Redox Biol..

[24] Zhang Z., Guo L., Yang F., Peng S., Wang D., Lai X., Su B., Xie H. (2023). Adiponectin Attenuates Splenectomy-Induced Cognitive Deficits by Neuroinflammation and Oxidative Stress via TLR4/MyD88/NF-κb Signaling Pathway in Aged Rats. ACS Chem. Neurosci..

[25] Li Q., Tang Y., Chen Y., Li B., Wang H., Liu S., Adeniran S.O., Zheng P. (2024). Melatonin Regulates the Expression of VEGF and HOXA10 in Bovine Endometrial Epithelial Cells through the SIRT1/PI3K/AKT Pathway. Animals.

[26] Guo Y., Sun J., Li T., Zhang Q., Bu S., Wang Q., Lai D. (2017). Melatonin ameliorates restraint stress-induced oxidative stress and apoptosis in testicular cells via NF-κB/iNOS and Nrf2/HO-1 signaling pathway. Sci. Rep..

[27] Pastorek M., Müller P., Vojtěšek B. (2015). Nrf2—Two Faces of Antioxidant System Regulation. Klin. Onkol..

[28] Fan Z., Wirth A.-K., Chen D., Wruck C.J., Rauh M., Buchfelder M., Savaskan N. (2017). Nrf2-Keap1 pathway promotes cell proliferation and diminishes ferroptosis. Oncogenesis.

[29] Magesh S., Chen Y., Hu L. (2012). Small molecule modulators of Keap1-Nrf2-ARE pathway as potential preventive and therapeutic agents. Med. Res. Rev..

[30] Kaspar J.W., Niture S.K., Jaiswal A.K. (2009). Nrf2, INrf2 (Keap1) signaling in oxidative stress. Free Radic. Biol. Med..

[31] Eizirik D.L., Flodström M., Karlsen A.E., Welsh N. (1996). The harmony of the spheres: Inducible nitric oxide synthase and related genes in pancreatic beta cells. Diabetologia.

[32] Estaras M., Gonzalez-Portillo M.R., Martinez R., Garcia A., Estevez M., Fernandez-Bermejo M., Mateos J.M., Vara D., Blanco-Fernández G., Lopez-Guerra D. (2021). Melatonin Modulates the Antioxidant Defenses and the Expression of Proinflammatory Mediators in Pancreatic Stellate Cells Subjected to Hypoxia. Antioxidants.

[33] Mi W., Yu M., Yin S., Ji Y., Shi T., Li N. (2022). Analysis of the Renal Protection and Antioxidative Stress Effects of Panax notoginseng Saponins in Diabetic Nephropathy Mice. J. Immunol. Res..

[34] Zhang Z., Li M., Wang Y., Wu J., Li J. (2014). Higenamine promotes M2 macrophage activation and reduces Hmgb1 production through HO-1 induction in a murine model of spinal cord injury. Int. Immunopharmacol..

[35] Qin T., Feng D., Zhou B., Bai L., Yin Y. (2022). Melatonin Suppresses LPS-Induced Oxidative Stress in Dendritic Cells for Inflammatory Regulation via the Nrf2/HO-1 Axis. Antioxidants.

